# Concurrent Typhoid Fever and Dengue Hemorrhagic Fever: A Case Report

**DOI:** 10.7759/cureus.28600

**Published:** 2022-08-30

**Authors:** Azeem Khalid, Asad Ur Rehman, Ahmadullah Baig, Waseh Ahsan, Muhammad Zaman Khan Assir

**Affiliations:** 1 Internal Medicine, Allama Iqbal Medical College/Jinnah Hospital, Lahore, PAK

**Keywords:** co-infection, enteric fever, salmonella, typhoid fever, dengue hemorrhagic fever

## Abstract

Dengue virus can co-infect with a number of viruses, bacteria, and parasites of which dengue malaria co-infection is most well-known. We report a rare case of dengue virus co-infection with typhoid fever and the development of dengue hemorrhagic fever (DHF) during a dengue outbreak. The second spike of high-grade fever following initial defervescence with antibiotic therapy, hemorrhagic manifestations, new onset leucopenia and thrombocytopenia, and evidence of plasma leakage raised suspicion of DHF. Diagnosis of dengue co-infection was made by seroconversion for anti-dengue immunoglobulin M (IgM) antibodies by enzyme-linked immunosorbent assay (ELISA) on the seventh day of new-onset fever. Early recognition and judicious use of fluid therapy prevented the patient from developing shock and its complications. Prompt diagnosis, early recognition of plasma leakage, and appropriate management of DHF can reduce morbidity and mortality.

## Introduction

Dengue fever is a fast-emerging pandemic-prone viral disease in many parts of the world. A recent study estimates the global burden of dengue to be 390 million infections per year, an infection total of more than three times the estimate of the World Health Organization [1]. With expanding geographical distribution of the disease, Pakistan has become hyper-endemic for dengue, and till 2016 almost 71649 dengue cases were reported with 757 deaths [1]. Dengue virus can coinfect other viruses, bacteria, and plasmodium [2]. Like many developing countries, Pakistan is also endemic to malaria and enteric fever. In Pakistan, dengue-malaria co-infection is relevant, but the same is not true for dengue-*Salmonella* co-infection [3]. Viral illnesses can be complicated by secondary bacterial infections and the dengue virus is no exception. In contrast with the epidemiological pattern noted in Pakistan, higher dengue-*Salmonella* co-infection (approximately four out of 9,997) have been reported [2]. Two cases of dengue hemorrhagic fever (DHF) complicated with *Salmonella *infection were reported from Indonesia [4]. Here we report a case of enteric fever complicated with dengue virus coinfection and subsequent development of DHF. To the best of our knowledge, this is the first case report of *Salmonella* infection complicated with DHF. 

## Case presentation

A 17-year-old girl presented to the medical emergency of a tertiary care hospital in Lahore, Pakistan during the dengue outbreak of November 2013. She had a history of low-grade fever for 15 days that was accompanied by generalized abdominal pain, vomiting, and constipation. She consulted her family physician and was worked up for possible enteric fever. Her complete blood counts and metabolic profile were normal and the ultrasound of the abdomen was unremarkable.

Her Typhidot immunoglobulin (Ig) M was positive and she was empirically treated with intravenous cefotaxime 1g q12hr for seven days. Blood cultures were not obtained. Her symptoms started to improve on the second day of antibiotic therapy. She had received *Helicobacter pylori *eradication therapy three months back for the endoscopic biopsy proven mild gastritis due to *H. pylori* infection. A Typhidot IgM advised at that time was negative. 

After initial defervescence on treatment with cefotaxime, she suddenly developed high-grade fever along with rigors and chills, myalgia, arthralgia, headache, retro-orbital pain, and mild gum bleeding. She also had complaints of abdominal pain and vomiting. She presented to us with four days history of new-onset high-grade fever. 

At presentation, she was febrile (102 degrees F) and tachycardic (pulse rate 100/min). Her blood pressure was 120/80 mmHg with a respiratory rate (RR) of 16/min. She had bilateral conjunctival hemorrhages. Her abdomen was soft but tender on deep palpation. There was no clinical evidence of organomegaly or ascites. The rest of the physical examination was unremarkable. Her labs revealed a recent sudden drop in leukocyte (3.1 × 109/L) and platelet counts (28 × 109/L). Her ultrasound abdomen and chest X-ray were unremarkable at presentation. Based on initial clinical signs and symptoms, she was hospitalized for further workup and management.

On the fifth day of illness and the first day of hospitalization, the patient developed a dry cough and mild dyspnoea at rest. She was febrile (101 degrees F) and tachypnoeic (RR of 24/min) with a stony dull percussion note and absent breath sounds over the right infra-scapular region. Her repeat X-ray chest and ultrasound abdomen revealed right-sided pleural effusion and mild pelvic ascites, respectively. The non-structural 1 (NS-1) antigen and dengue-specific IgM by enzyme-linked immunosorbent assay (ELISA) were negative on day five. She had seroconversion for dengue-specific IgM on the seventh day. 

Anti-dengue IgM antibodies were measured by indirect IgM ELISA using the commercially available kit (Human GmbH, Wiesbaden, Germany). The ELISA for indirect IgM antibody detection uses dengue-specific antigens (DEN-Ag) coated on microtitre wells. The IgM titer on day seven was 0.827 (cut-off value of 0.424). Peripheral blood film and rapid diagnostic tests were negative for malarial parasites. The patient became afebrile on the seventh day of illness. Owing to her persistent tachycardia, her cardiac enzymes and echocardiography were ordered to rule out myocarditis which turned out to be normal. The patient was treated with acetaminophen, intravenous crystalloids, and intravenous cefotaxime. For the management of DHF, maintenance fluid therapy was initiated at around 100 ml/hr. After eight to 10 hours, the patient received a fluid bolus of 500 ml over one hour as her tachycardia was worsening and pulse pressure was becoming narrower. The fluid bolus resulted in her clinical improvement and the fluid therapy was gradually reduced to 50 ml to 75 ml/hr over the next few hours. The patient received 2200 ml out of a total fluid quota of 4600 ml. After five days of in-hospital monitoring and management, the patient recovered, the pelvic ascites and right-sided pleural effusion resolved and the patient was discharged. 

## Discussion

The clinical spectrum of dengue infection varies from asymptomatic infection to life-threatening dengue shock syndrome. Dengue hemorrhagic fever is distinct from dengue fever due to the presence of plasma leakage. It results from sequential infection by two different viral serotypes [5].

Enteric fever is characterized by persistent fever, malaise, and GI complaints and may cause a protracted illness lasting several weeks. The disease is seldom fatal, but some patients can develop life-threatening complications, including hypotensive shock and intestinal perforation [6].

The patient in this report was diagnosed with a case of enteric fever based on a fever of 15 days duration, abdominal complaints, positive Typhidot IgM, and initial response to injectable antibiotics. A negative Typhidot IgM two months back implies a recent infection with *Salmonella*. Typhidot qualitatively detects the presence of IgM and IgG antibodies to a 50kDa outer membrane protein. In different population-based typhoid surveillance studies in several countries, the sensitivity and specificity for Typhidot were around 70% and 80%, respectively [7,8]. The definitive diagnosis of enteric fever can be established by obtaining growth of* Salmonella* on blood, stool, or bone marrow cultures. In our case, a definitive diagnosis could not be established as blood cultures were not advised before the initiation of antibiotics. 

A sudden change in the character of the fever with new onset constitutional symptoms, bleeding and bi-cytopenia in the setting of an established dengue outbreak prompted us to investigate for possible dengue infection. The diagnosis of DHF is based on four criteria: fever, bleeding tendency, thrombocytopenia (platelet count < 100,000/mm3), and evidence of plasma leakage (rise in hematocrit of ≥20% from baseline, pleural effusion or ascites). The specificity of the dengue hemorrhagic fever (DHF) case definition implies that a diagnosis of DHF does not require laboratory evidence of dengue virus infection [9]. 

The diagnosis of dengue viral infection is confirmed by viral isolation, reverse transcription polymerase chain reaction (RT-PCR), dengue viral NS-1 antigen, seroconversion from negative to positive IgM antibody to dengue, or demonstration of a fourfold or greater increase in IgG antibody titers in paired (acute and convalescent) serum specimens [5]. Our patient had seroconversion for dengue-specific IgM on day seven, which was highly suggestive of a new dengue infection. The NS-1 antigen was negative in our patient. The diagnostic sensitivity of NS-1 antigen in primary dengue infection during the febrile phase may exceed 90% but it is lower in secondary infections (60 to 80%) [10]. Due to the increasing resistance of* Salmonella *to quinolones, our patient received an injection of cefotaxime 1g intravenously for the treatment of enteric fever. 

## Conclusions

Concurrent infection of dengue fever can occur with other endemic diseases like typhoid fever. The changing pattern of fever in the setting of a dengue outbreak should raise the suspicion of co-infection with dengue virus. Prompt diagnosis, early recognition of plasma leakage and appropriate management of DHF can reduce morbidity and mortality. 
